# A subfamily roadmap of the evolutionarily diverse glycoside hydrolase family 16 (GH16)

**DOI:** 10.1074/jbc.RA119.010619

**Published:** 2019-09-09

**Authors:** Alexander Holm Viborg, Nicolas Terrapon, Vincent Lombard, Gurvan Michel, Mirjam Czjzek, Bernard Henrissat, Harry Brumer

**Affiliations:** ‡ Michael Smith Laboratories, University of British Columbia, Vancouver, British Columbia V6T 1Z4, Canada; § Architecture et Fonction des Macromolécules Biologiques, CNRS, Aix-Marseille Université, F-13288 Marseille, France; ¶ USC1408 Architecture et Fonction des Macromolécules Biologiques, Institut National de la Recherche Agronomique, F-13288 Marseille, France; ‖ Sorbonne Universités, CNRS, Integrative Biology of Marine Models (LBI2M), Station Biologique de Roscoff, 29680 Roscoff, France; ** Department of Chemistry, University of British Columbia, Vancouver, British Columbia V6T 1Z1, Canada; ‡‡ Department of Biochemistry and Molecular Biology, University of British Columbia, Vancouver, British Columbia V6T 1Z3, Canada; §§ Department of Botany, University of British Columbia, Vancouver, British Columbia V6T 1Z4, Canada; ¶¶ Department of Biological Sciences, King Abdulaziz University, Jeddah 21589, Saudi Arabia

**Keywords:** phylogenetics, enzyme structure, protein evolution, structural biology, glycoside hydrolase, beta-jelly-roll fold, beta-sandwich, carbohydrate-active enzymes (CAZymes), Hidden Markov Model (HMM), sequence similarity networks (SSN)

## Abstract

Glycoside hydrolase family (GH) 16 comprises a large and taxonomically diverse family of glycosidases and transglycosidases that adopt a common β-jelly-roll fold and are active on a range of terrestrial and marine polysaccharides. Presently, broadly insightful sequence–function correlations in GH16 are hindered by a lack of a systematic subfamily structure. To fill this gap, we have used a highly scalable protein sequence similarity network analysis to delineate nearly 23,000 GH16 sequences into 23 robust subfamilies, which are strongly supported by hidden Markov model and maximum likelihood molecular phylogenetic analyses. Subsequent evaluation of over 40 experimental three-dimensional structures has highlighted key tertiary structural differences, predominantly manifested in active-site loops, that dictate substrate specificity across the GH16 evolutionary landscape. As for other large GH families (*i.e.* GH5, GH13, and GH43), this new subfamily classification provides a roadmap for functional glycogenomics that will guide future bioinformatics and experimental structure–function analyses. The GH16 subfamily classification is publicly available in the CAZy database. The sequence similarity network workflow used here, SSNpipe, is freely available from GitHub.

## Introduction

Complex carbohydrates—oligosaccharides and polysaccharides of diverse residue and linkage composition—are central to a wide range of biological processes, such as energy storage, inflammation, host–pathogen interactions, diseases, and differentiation/development (1). Not least, manifold complex carbohydrates play essential structural roles in the cell walls in terrestrial and marine biomass (2, 3).These biomass sources represent major sinks in the global carbon cycle (4, 5) and a vast renewable resource for the production of energy, chemicals, and materials (6).

The synthesis, rearrangement, and ultimate saccharification of the vast diversity of glycosidic linkages in natural carbohydrates require a correspondingly broad range of specific carbohydrate-active enzymes (CAZymes).^4^ In light of the continually accelerating rate of sequence data deposition, the CAZy database has emerged as a central resource uniting specificity, mechanistic, and structural information within actively curated, sequence-based families of glycosyltransferases, glycoside hydrolases (GHs), polysaccharide lyases, carbohydrate esterases, auxiliary activity enzymes, and associated noncatalytic carbohydrate-binding modules (CBMs) (7, 8). The CAZy classification offers extraordinary predictive power on the family level, whereby the key active-site residues, the catalytic mechanism, and the overall three-dimensional fold are generally strictly conserved. Family classification is also a broad predictor of substrate specificity, in terms of overall glycosidic linkage orientation (α or β) and saccharide composition. However, the subtle natural variations in configuration among structurally related groups of complex carbohydrates has given rise to several “polyspecific” families, which comprise diverse activities. As it pertains to genomics and bioinformatics, polyspecificity confounds precise functional annotation of CAZyme family members in the absence of biochemical data (7).

The problem of polyspecificity is especially significant among large CAZyme families, which may encompass tens of thousands of sequences from taxonomically diverse organisms. In such cases, division into subfamilies based on molecular phylogeny has been shown to significantly increase predictive power in a handful of GH and polysaccharide lyase families previously (9–13). However, a major limitation of large-scale phylogenetic analyses is the dependence on a highly accurate multiple sequence alignment (MSA) (14) and subsequent phylogenetic tree estimation, in which the computational complexity increases exponentially with the number of sequences (15). As the number of nonredundant sequences in the CAZy database increases (7), highly accurate subfamily phylogenies will be infeasible for most families in the foreseeable future.

**Figure 1.**
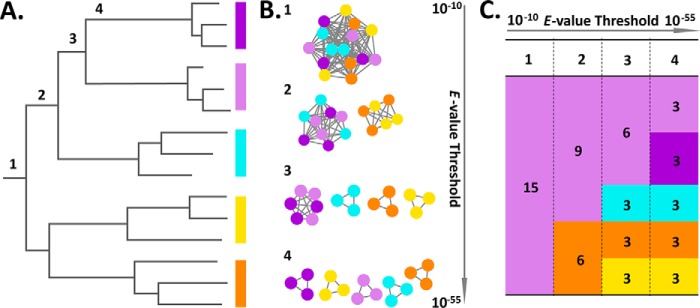
**Subfamily delineation based on distinct analysis/representation.** This artificial example of 15 sequences to be classified into subfamilies illustrates the relationships between distinct representation and analysis. The *numbers 1–4* indicate four hypothetical subfamily classifications that are concordant in all three representations. *A*, evolutionary tree. Reconstruction from a phylogenetic analysis or hierarchical clustering. Subfamily delineation consists of drawing a *vertical line* (below *numbers 1–4*) and making a family for each outcoming branch. *B*, SSN connection graph. SSNs with sequences represented as nodes (*circles*) and all pairwise sequence relationships (alignments) above a defined *E*-value threshold indicated with edges (*lines*). At increased thresholds (*numbers 1–4*), the connected components break up into an increasing number of subcomponents, representing putative subfamily delineations (16). *C*, SSN tabular summary. Each column (*numbers 1–4* for each *E*-value threshold, separated by a vertical *dashed line*) depicts a distinct subfamilization and displays the number of clusters/subfamilies as *colored boxes* and the number of members/sequences in each cluster/subfamily.

Sequence similarity networks (SSNs), which are conceptually illustrated in Fig. 1, offer a potential solution to this conundrum. In contrast to MSA-based phylogenies, SSNs are based on all-*versus*-all pairwise local sequence alignments, the computational requirements of which scales linearly with the number of sequences and are easily amenable to parallelization. Notably, the resulting networks of nodes and edges, which can be rapidly generated using any Expect (*E*) value or bit score as a threshold, usually resolve the same monophyletic groups observed in corresponding phylogenetic trees (16). Like phylogenetic approaches, SSNs can underpin the creation of subfamilies and establish a robust framework to predict substrate specificity and highlight unexplored sequence space (17).

Glycoside hydrolase family 16 (GH16) is a polyspecific family of β-glycanases involved in the degradation or remodeling of cell wall polysaccharides in marine and terrestrial biomass (see Table 1). GH16 represents a current challenge for functional subfamily classification because of its large size and diversity. GH16 members are widely distributed across the domains of life, including bacteria (18), oomycetes (19), fungi (20, 21), plants (22, 23), and animals (terrestrial insects and marine invertebrates (24, 25)), in which they play manifold biological roles. GH16 members are united by a compact (∼30 kDa) β-jelly roll structural fold (26), which nonetheless has a remarkable evolutionary plasticity that gives rise to specificities for a plethora of complex terrestrial and marine cell-wall carbohydrates, hydrolase and transglycosylase activities, and noncatalytic substrate-binding functions (21, 27–29). Presently, GH16 comprises ∼8,000 sequences in the public CAZy database representing 15 known activities (7), which is comparable with other large families (GH5 and GH43) for which subfamily classifications have been established (GH13 is an exception, with nearly 10-fold more members, whereas GH30 is 4-fold smaller than GH16) (9–13). Only 2.5% of GH16 sequences have been enzymatically characterized (7), which challenges functional prediction.

Here we present a comprehensive subfamily classification of GH16 based on large-scale SSN analysis of the entire GH16 sequence space as a roadmap for future functional glycogenomics. The subfamily topology was equal to that obtained by classical phylogenetic analysis of a reduced sequence data set. The resulting robust subfamilies were used in turn to generate hidden Markov models (HMMs), which will form the basis for the automated incorporation of new sequences into the continually expanding CAZy database.

## Results

### Subfamily delineation

**Figure 2.**
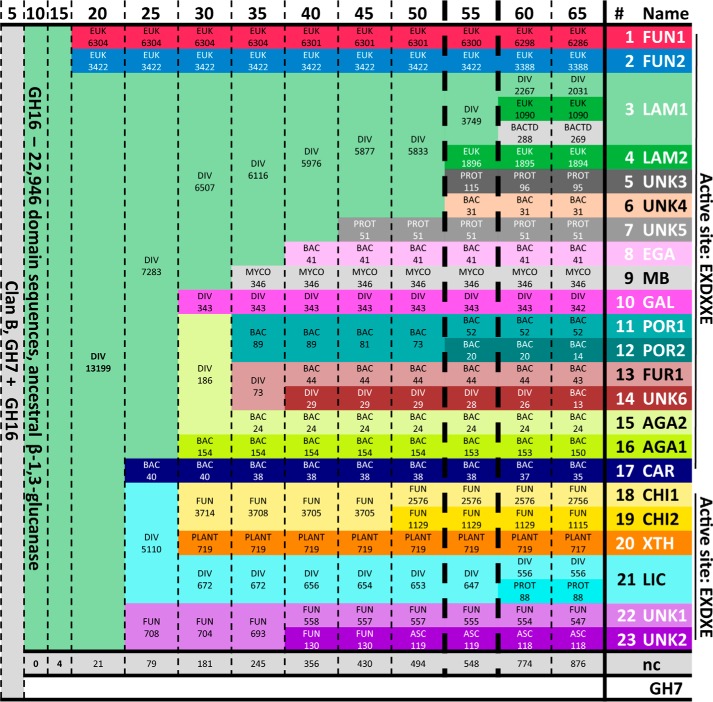
**Summary of GH16 sequence similarity networks.** Summary of the subfamilies created in SSNs under thresholds from *E* = 10^−5^ to 10^−65^. The *top row* indicates the SSN clustering threshold defining each column (*e.g.* “35” corresponds to an *E*-value threshold of 10^−35^). The *rows* represent the emergent subfamilies (colored individually) and their stability across thresholds. Labels in the subfamilies indicate the number of sequence members as well as the taxonomic range. *ASC*, Ascomyocota; *BAC*, Bacteria; *BACTD*, Bacteroidetes; *DIV*, multiple kingdoms; *EUK*, Eukaryota; *FUN*, fungi; *MYCO*, *Mycobacterium*; *PLANT*, Plantae; *PROT*, Proteobacteria. Definitive subfamilies defined based on the *E* = 10^−55^ threshold (column marked with *bold dashed lines*) are numbered in the *right-most column*, in ascending order according to the family size/sequence members. Subfamily mnemonics assigned based on known activities or taxonomic distribution are as follows: *AGA*, β-agarases; *CAR*, κ-carrageenase; *CHI*, chitin β(1,6)-glucanosyltransferase; *EGA*, *endo*-β(1,4)-galactosidases; *FUN*, fungal; *FUR*, Furcellaranase; *GAL*, *endo*-β(1,3)-galactanases; *LAM*, *endo*-β-glucanases; *LIC*, *endo*-β(1,3)/β(1,4)-glucanase; MB, Mycobacterium; *POR*, β-porphyranases; *UNK*, Unknown; *XTH*, Xyloglucan *endo*-tranglycosylase/*endo*-hydrolase. The *bottom row* show the nonclassified (*nc*) sequences, not assigned to any subfamily (548 of 22,946 total GH16 sequences at the 10^−55^ threshold).

All-*versus*-all pairwise local sequence alignments were calculated for 22,946 GH16 domain sequences from the CAZy database in 210 min on a desktop computer (Intel Xeon Processor E5-1620 v4, 8 cores, 3.5 GHz, 16 GB RAM). For comparison, the computational time was reduced to 13 min using 128 cores on Compute Canada's WestGrid high-performance infrastructure. Subsequently, the BLAST result file was indexed over thresholds in intervals of 5 log units for *E* values between 10^−5^ and 10^−120^. Our preliminary SSN and HMM analyses indicated that the 10 SSNs for *E-*value thresholds between 10^−20^ and 10^−65^ were of most interest with the number of subfamilies ranging from 3 at *E* = 10^−20^ to 27 at *E* = 10^−65^ (Fig. 2). Mapping sequence origin and the 15 currently known substrate specificities (from nearly 200 biochemically characterized GH16 proteins (7) (Table 1) reveals the distribution of these features across emergent subfamilies (Fig. 2).

**Table 1:** **Defined subfamilies within GH16**

	Name	Taxonomical distribution	EC		No. of sequences	No. of characterized members	Representative PDB structure (reference)
1	FUN1	Eukaryota	3.2.1.39	*Endo*-β (1,3)-glucanase	6,300	13	2CL2 (33)
			3.2.1.6	*Endo*-β (1,3)/β (1,4)-glucanase			
			3.2.1.35	Hyaluronidase			
			2.4.1.–	Transglycosylase			
2	FUN2	Eukaryota	2.4.1.–/3.2.1.–	Transglycosylase	3,422	1	
3	LAM1	Diverse	3.2.1.39	*Endo*-β (1,3)-glucanase	3,749	38	4CTE (37)
			3.2.1.6	*Endo*-β (1,3)/β (1,4)-glucanase			
4	LAM2	Eukaryota	3.2.1.39	*Endo*-β (1,3)-glucanase	1,896	13	
5	UNK3	Proteobacteria			115	0	
6	UNK4	Bacteria			31	0	
7	UNK5	Proteobacteria			51	0	
8	EGA	Bacteria	3.2.1.–	*Endo*-β (1,4)-galactosidase	41	1	
9	MB	*Mycobacterium*			346	0	4PQ9 (unpublished)
10	GAL	Diverse	3.2.1.181	*Endo*-β (1,3)-galactanase	343	3	
11	POR1	Bacteria	3.2.1.178	β-Porphyranase	52	1	3JUU (39)
12	POR2	Bacteria	3.2.1.178	β-Porphyranase	20	3	4AWD (40)
13	FUR1	Bacteria	3.2.1.–	Furcellaranase	44	1	
14	UNK6	Diverse			28	0	
15	AGA2	Bacteria	3.2.1.81	β-Agarase	24	2	6HY3 (42)
16	AGA1	Bacteria	3.2.1.81	β-Agarase	153	32	4ATF (43)
17	CAR	Bacteria	3.2.1.83	κ-Carrageenase	38	6	5OCR (44)
18	CHI1	Fungi	2.4.1.–	Chitinβ (1,6)-glucanosyltransferase	2,576	2	6IBW (unpublished)
			2.4.1.–/3.2.1.–	Cell-wall modifying			
19	CHI2	Fungi	2.4.1.–	Chitinβ (1,6)-glucanosyltransferase	1,129	1	
20	XTH	Plantae	2.4.1.207	Xyloglucan *endo*-tranglycosylase	719	34	2VH9 (48)
			3.2.1.151	Xyloglucan *endo*-hydrolase			
21	LIC	Diverse	3.2.1.73	*Endo*-β (1,3)/β (1,4)-glucanase	647	35	1GBG (54)
22	UNK1	Fungi			555	0	
23	UNK2	Ascomycota			119	0	

**Figure 3.**
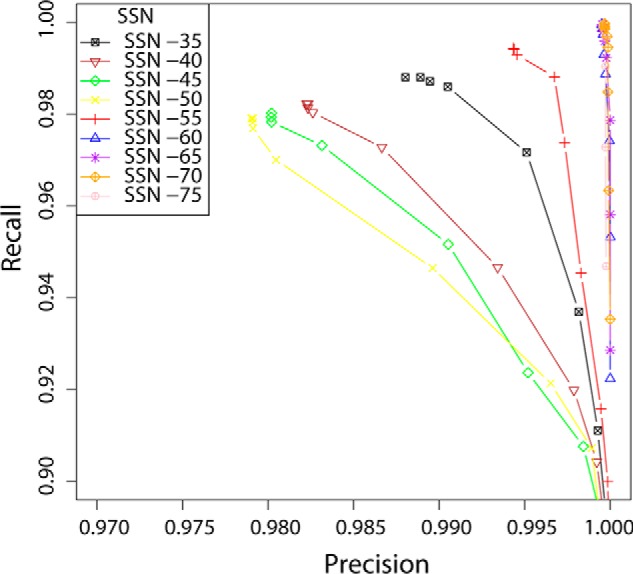
**Performance of GH16 hidden Markov model libraries.** HMM libraries of GH16 subfamilies, generated from the SSN at each threshold (color-coded in the legend), were evaluated in their ability to assign each GH16 module to the correct subfamily delineated by the individual SSNs. The *curves* show the evolution of the precision and recall (see “Experimental Procedures” for definitions) with increasing SSN *E*-value cutoff (*cf.* Fig. 2 and Fig. 4), with points corresponding to variation in HMM *E*-value thresholds.

To determine the threshold at which optimal discrimination of subfamilies is achieved, a library of HMMs was created for each SSN, and their performance was evaluated by computing precision and recall rates using all 22,946 GH16 members as input (Fig. 3). It was observed that at a threshold of *E* = 10^−60^, the HMM library was able to retrieve all of the sequence assignments into the 26 subfamilies, with limited loss of precision at high *E* values, compared with SSN based on lower thresholds (Fig. 3). For SSNs induced by higher thresholds, GH16 was only broken down into an increasing number of subfamilies, primarily along taxonomic lines (Figs. S1 and S2). Such divisions are unlikely to be functionally significant and rather are likely only to reflect sequence drift caused by speciation. In this analysis, it is also helpful to keep the limit analysis in mind: division of GH16 into 22,946 individual subfamilies would result in recall and precision values of 100% at the subfamily level, yet it would provide no predictive power. Thus, although the data in Fig. 3 would suggest that the HMM library from the SSN at *E* = 10^−60^ may have the best performance, practically this represents little performance gain and might be unnecessarily stringent. Analysis of the taxonomic distribution and number of unclustered sequences between the SSN at *E* = 10^−60^ and the previous SSN at *E* = 10^−55^ suggest that the latter would be a more pragmatic choice, considering that the continuous growth of GH16 family would likely result in new sequences filling the gaps between subfamilies that are too finely divided. Hence, the SSN at *E* = 10^−55^ and the corresponding HMM library was chosen for the creation of the final subfamilies in GH16.

**Figure 4.**
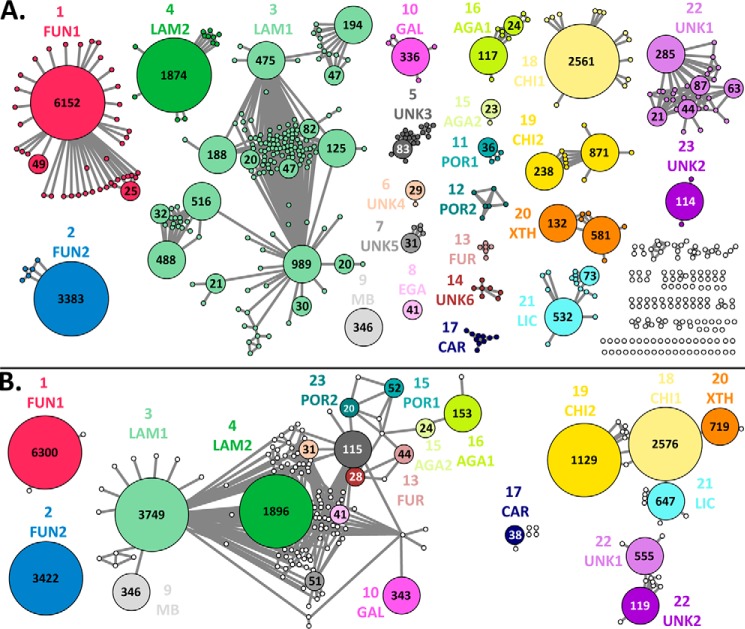
**Sequence similarity networks of 22,946 GH16 sequences.** *A*, edges represent an *E*-value threshold below 10^−55^. Metanodes represent highly similar sequences (*E* > 10^−85^); only metanodes containing 20 or more sequences are enlarged, with the number of merged sequences indicated. The network defines 23 subfamilies (see Fig. 2 for subfamily numbering and mnemonics). Clusters that lack sufficient taxonomic diversity or size to define subfamilies are indicated in *white. B*, edges represent an *E*-value threshold below 10^−25^. Metanodes represent defined subfamilies in *A* (*E* > 10^−55^); the network displays the basic relationship of subfamilies at this relaxed threshold (*cf.* Fig. 2).

In total, 23 subfamilies were defined using the SSN based on the *E* = 10^−55^ threshold (Fig. 4*A*), which collectively assigned 22,367 sequences to a subfamily (97.5% of all GH16 modules analyzed). Subfamily size ranges from 20 to 6,300 sequences. The taxonomical diversity within subfamilies mainly occurs at the phylum level, with only four subfamilies (GH16_3, GH16_10, GH16_14, and GH16_21) present in multiple kingdoms of life. The lowest taxonomic diversity was in an early diverging group of mycobacterial sequences (GH16_9), which robustly formed a distinct subfamily (Fig. 2). Notably, one of the earliest emerging features that distinguishes subfamilies is the presence or absence of the β-bulge sequence motif (E*X*D*XX*E *versus* E*X*D*X*E) in the active-site β-strand presenting the catalytic residues (Fig. 2), which is a key structural feature among GH16 members (30).

**Figure 5.**
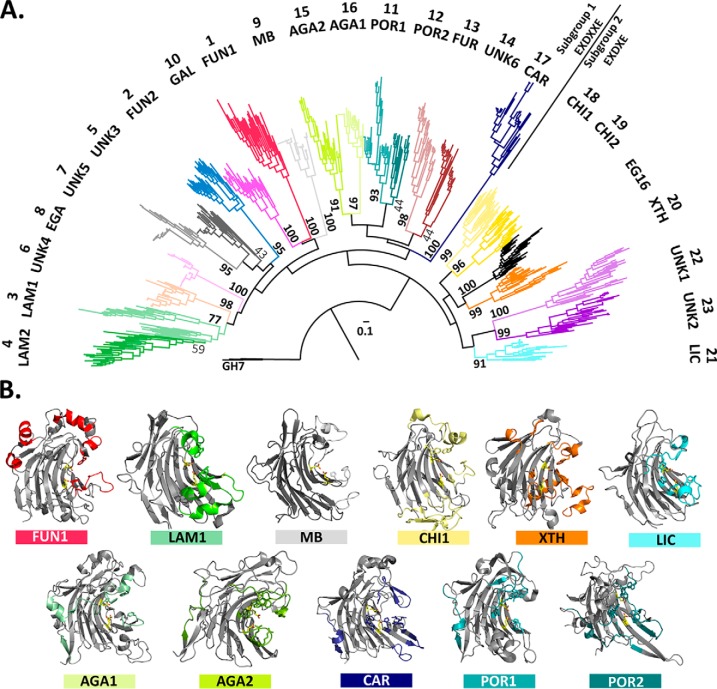
**Phylogenetic tree and structure–function relationships of GH16.** *A*, maximum-likelihood phylogenetic tree was generated using up to 30 representative sequences for each GH16 subfamily defined by the sequence similarity network shown in Fig. 4. Three GH7 cellulases (GH7 and GH16 constitute clan GH-B) (7) were used to root the tree. Bootstrap values based on 100 replicates are shown. The tree separates (indicated by a *line*) GH16 enzymes with the β-bulge active-site motif E*X*D*XX*E from those with the β-strand active-site motif E*X*D*X*E, concordant with previous analyses (30, 51). Branch coloring is identical to that used in Figs. 2 and 4; subfamily numbering and mnemonics are given in Fig. 2. Subfamily membership of all GH16 members is available on the actively curated CAZy database (http://www.cazy.org/GH16.html).^5^ *B*, ribbon drawings of 3D structures of representative subfamily members (where present, see Table 1). *Loops*, structural elements and residues that are characteristic of a given subfamily are colored with their respective color (*color bar* underneath the structural icon), the same as in the phylogenetic tree in *A*.

A limitation of SSNs is the inability to establish phylogenetic relationships between subfamilies. To establish overall context and to validate further the subfamily classification of GH16, a maximum-likelihood phylogenetic tree was constructed from 30 randomly selected sequences from each subfamily defined by the SSN. The delineation of subfamilies from the SSN (Fig. 2 and Fig. 4*A*) is identical to the monophyletic groups inferred from the phylogenetic tree (Fig. 5*A*). Importantly, all clades comprising individual subfamilies are supported by high bootstrap values.

The SSN analysis delineated GH16 sequences into “characterized” subfamilies with one or more biochemically or structurally characterized members (denoted in the CAZy database (7)) and “uncharacterized” subfamilies for which structural–functional data are currently lacking. Table 1 summarizes the taxonomic range of source organisms, experimentally determined enzyme activities, and available tertiary structures for each subfamily shown in Fig. 4*A*. Specific sequence accessions, including subfamily membership and characterization details, may be accessed directly in the CAZy database (http://www.cazy.org/GH16.html) (7).^5^ In total, 16 of 23 subfamilies contained at least one biochemically characterized member, and 11 had a three-dimensional structure representative. Salient features of individual subfamilies are detailed below. Analogous to previous GH subfamily classifications (9, 10, 12), subfamilies are systematically referenced as “GH16_*n*,” where *n* is the subfamily number.

### Characterized subfamilies

#### 

##### GH16_1

The largest GH16 subfamily, GH16_1, has 6,300 members, which comprise almost exclusively fungal enzymes, with a few members from a pathogenic nematode. GH16_1 is very distinct, already separating at a threshold of *E* = 10^−20^ and exhibiting no significant segregation prior to a threshold of *E* = 10^−85^ (Fig. 4*A* and Fig. S1). Only fungal enzymes have been characterized in this subfamily: *endo*-β(1,3)-glucanases (EC 3.2.1.39) and *endo*-β(1,3)/β(1,4)-glucanases (EC 3.2.1.6) have been reported for nine enzymes, whereas activity toward hyaluronate (hyaluronidase, EC 3.2.1.35) has been reported for two enzymes (31). Interestingly, one representative of this subfamily has been reported to be an *exo*-β(1,3)-glucosyltransferase/elongating β-transglucosylase (EC 2.4.1.–) (32).

Structurally, GH16_1 is defined by the presence of numerous helical elements on the core β-jelly-roll fold: two in the N-terminal region and four in the C-terminal region, most of which are located on the opposite side of the structure from the active site cleft (Fig. 5*B*). The α5 helix carries a conserved tryptophan, Trp-257 (PDB code 2CL2 (33)), that points into the active site and faces a loop, which is consolidated by a disulfide bridge. Together, these elements define the positive enzyme subsites (34) in this subfamily. A notable sequence pattern “WPA … WP*X*” (*X* is often Y or N, but also A, T, or I) is shared with GH16_3 and GH16_9 members. The WP*X* motif is located in a loop bordering the active-site cleft at the negative subsites and therefore likely contributes to substrate specificity.

##### GH16_2

Members of GH16_2 are almost exclusively reported in fungi, with less than 2% of the members found in plant-damaging oomycetes (water molds) and algae. GH16_2 is very distinct and shows almost no sequence diversity even at a threshold of *E* = 10^−120^ (Figs. S1 and S2). Only a single biochemically characterized member, a cell-wall active β(1,6)-glucanase/transglucosylase (EC 3.2.1.–/2.4.1.–) from *Saccharomyces cerevisiae*, is known (35). Interestingly, GH16_2 members lack a signal peptide that is otherwise commonly associated with members of fungal GH16 subfamilies. No tertiary structural representatives currently exist in GH16_2.

##### GH16_3

Historically known as the laminarinase subfamily (30, 36), GH16_3 is a large and extremely sequence-diverse subfamily (Fig. 4*A*) found in all kingdoms. *Endo*-β(1,3)-glucanase (EC 3.2.1.39) and/or *endo*-β(1,3)/β(1,4)-glucanase activity (EC 3.2.1.6) has been reported in members of the Metazoa, Fungi, Archaea, and Bacteria. The broad taxonomical diversity of GH16_3 members makes this subfamily particularly sensitive to the threshold *E*-value cutoff, such that increasingly strict cutoff values result in fragmentation along taxonomic lines.

The large sequence and taxonomic diversity is reflected by low structural homology in this subfamily, where only very few stretches and features are strictly conserved among the subfamily members. However, the sequence pattern “WPA … W*XX* … WP*X*” (*X* being M or L for the second motif and A, K, R, M, or L for the third motif), similar to that found in GH16_1, is largely conserved throughout members of this subfamily. In GH16_3, this loop faces a short, subfamily-specific π-helical element that is located in the N-terminal region (residues 25 to 34 in PDB code 4CTE (37)). Furthermore, a tryptophan or phenylalanine that lines the active site in the positive subsites is part of a partially conserved motif present in many subfamily members, as is a loop (His-155 to His-163) that contains a strongly conserved histidine residue (His-155) facing this aromatic side chain. A structurally conserved short helical segment in different GH16_3 members (residues 210–218) is located next to this loop and possibly participates in shaping the overall active-site cleft of GH16_3.

##### GH16_4

GH16_4 can be considered as a subfamily derived from GH16_3, which segregates along with GH16_5 and GH16_6 at lower *E*-value thresholds (Figs. 2 and 4*B*). GH16_4 contains members from the Metazoa and Fungal kingdoms, with *endo*-β(1,3)-glucanase (EC 3.2.1.39) activity reported for 13 enzymes from Metazoa. Significantly, ∼9% of the 1,900 GH16_4 members, across Metazoa and Fungi, have lost one or both of their catalytic residues, although this feature is not resolved into monophyletic groups in a phylogenetic analysis (data not shown). In comparison, this is the case for only 0.7% of GH16_3 members and 1% of all other GH16 members. No tertiary structural representatives currently exist in GH16_4.

##### GH16_8

One enzyme in the GH16_8 subfamily has been demonstrated to have *endo*-β(1,4)-galactosidase activity (EC 3.2.1.–) (38). The members of this subfamily have very high sequence similarity (no fragmentation in the SSN from *E* = 10^−40^ to 10^−120^, Fig. 2, Figs. S1 and S2), despite having members from both Firmicutes and Actinobacteria. Approximately 75% of GH16_8 enzymes are linked to CBM32, members of which are known to bind galactose and are associated with wide variety of other GH domains. No tertiary structural representatives currently exist in GH16_8.

##### GH16_9

GH16_9 is comprised entirely of members from *Mycobacteria*. Although this observation contravenes our usual strict requirement for taxonomic diversity to establish a subfamily, the early segregation of this group at comparatively high *E* values (Fig. 2) supports the creation of a robust subfamily. Presently, biochemical function has not been defined for any GH16_9 member, but five members have been structurally characterized.

A structural characteristic of this subfamily is the low content of helical elements (Fig. 5*B*), in which only a short helix is present in the N-terminal region adjacent to the first loop near the negative subsites. Remarkably, GH16_9 members generally lack aromatic residues in the negative subsites as compared with other subfamilies. A tryptophan (Trp-154 in PDB code 4PQ9 (unpublished)) present in a conserved loop is positioned to accommodate a substrate in the positive subsites. Additionally, a conserved histidine (His-161), which is also present in GH16_1, GH16_3, GH16_16, GH16_11, GH16_17, and GH16_12, is found on the β-strand next to the catalytic E*X*D*XX*E motif.

##### GH16_10

*Endo*-β(1,3)-galactanases are exclusive to subfamily GH16_10, members of which have very high sequence similarity (SSN analysis indicates a stable group until a threshold cutoff of *E* = 10^−85^; Fig. 4*A*). Strikingly, this similarity is maintained across a wide taxonomic diversity, including the bacterial phyla Actinobacteria and Bacteroidetes, the fungal phyla Ascomycota and Basidiomycota, and the early-diverging fungal lineage Chytridiomycota. *Endo*-β(1,3)-galactanase activity has been reported twice in Ascomycota species and once in Basidimycota, whereas the bacterial members remain to be biochemically characterized. No tertiary structural representatives currently exist in GH16_10.

##### GH16_11

GH16_11 is composed exclusively of bacterial members from the phylum Bacteroidetes, except for one member from *Coraliomargarita*, a bacterial member of the phylum Verrucomicrobia. The activity in GH16_11 is defined based on a single biochemically characterized β-porphyranase (EC 3.2.1.178) (39).

Some key structural features of GH16_11 are shared with the β-agarase (GH16_15 and GH16_16), the β-porphyranase (GH16_12), and the κ-carrageenase (GH16_17) subfamilies, which is consistent with their close phylogenetic relationships (Fig. 5*a* and Fig. S3). These subfamilies have a characteristic N-terminal feature that consists of a short β-strand followed by a helical element, which is not present in other GH16 members. GH16_11 is distinguished further by the spatial organization of the first loop bordering the negative subsites of the active-site cleft, as well as a conserved loop close to the C terminus. This loop contains a characteristic arginine residue (Arg-70 in PDB code 3JUU (39)) in addition to a conserved tryptophan, Trp-67, that is also present in GH16_16, both of which are involved in substrate binding. The loop formed by a conserved sequence motif close to the C terminus (residues 256–265 in PDB code 3JUU) is also structurally distinct from those in other subfamilies.

##### GH16_12

Like GH16_11, GH16_12 is composed exclusively of bacterial members from the Bacteroidetes phylum, except for one member from *Coraliomargarita* (Verrucomicrobia). GH16_12 contains three biochemically characterized β-porphyranases (EC 3.2.1.178). GH16_11 and GH16_12 are highly related and form a uniform subfamily at lower thresholds, precisely resolving into two subfamilies at the SSN threshold of *E* = 10^−55^ (Figs. 2 and 4).

Consistent with the high relatedness of the two subfamilies, the major characteristic structural features are shared between the two subfamilies, including the N terminus and the first loop bordering the negative subsites. GH16_12 is distinguished by specific amino acid substitutions in the aromatic platform of the −1 subsite, as well as various loops throughout the tertiary structure. Specifically, a loop between the C-terminal two β-strands shared with GH16_11 is distinguished by sequence motives that are not identical between the two subfamilies, namely the stretch from residues 221 to 230 is WNPVPKDGGM in PDB code 3JUU, whereas the structurally identical stretch from residues 288 to 297 is WEKQVPTAED in PDB code 4AWD (40). Additionally, the motif comprising residues 210 to 228 in 4AWD, which in many other subfamilies forms a β-strand that terminates the β-sheet at the positive subsites, has a characteristic structure in GH16_12 members. This structure begins at the level of the inner concave β-sheet at the positive subsites and then changes level to spatially board the outer β-sheet of the β-jelly-roll fold.

##### GH16_13

GH16_13 comprises sequences from marine bacteria and is the newest subfamily to have its activity revealed by biochemical characterization. One biochemical characterized member was shown to hydrolyze furcellaran, a hybrid carrageenan containing both β-carrageenan and κ/β-carrageenan motifs (41). This subfamily has wide taxonomic distribution in the bacterial kingdom. No tertiary structural representatives currently exist in GH16_13.

##### GH16_15

Two β-agarases (EC 3.2.1.81) have been reported in the small GH16_15 (currently 24 members). This subfamily is very distinct from the other β-agarase-containing subfamily, GH16_16 (Fig. 2), to which it forms a sister clade with high bootstrap support (Fig. 5*A*). A member of GH16_15 has recently been shown to hydrolyze specifically complex agars from *Ceramiales* species, functionally distinguishing this subfamily from GH16_16 (42). Notably, unlike GH16_16, no CBMs are associated with GH16_15.

Together with functional characterization, the first structural representative of GH16_15 has recently been solved (PDB code 6HY3) (42). This structure reveals high structural similarity with GH16_16, with differences mainly observed in specific amino acid substitutions. Particularly notable are two aromatic residues (Trp-110 and Tyr-112 in PDB code 6HY3) that are located in the negative binding subsites and a characteristic loop (residues 291–300) located near the positive binding subsites, which presents two tryptophan residues (Trp-291 and Trp-297) that point into the active-site cleft. Another unique feature of GH16_15 is the presence of a conserved arginine (Arg-186) near the active site E*X*D*XX*E motif, as well as a second strictly conserved arginine (Arg-224) located in the positive subsites.

##### GH16_16

Considering the size of GH16_16 (153 sequences), it is the most densely studied subfamily in GH16 with 32 biochemically characterized β-agarases (EC 3.2.1.81) from Bacteroidetes, Proteobacteria, and Actinobacteria. A CBM13 or CBM6 is found associated with approximately half of the GH16_16 members.

In GH16_16 a characteristic N terminus is followed by an α-helix (Gly-94–Glu-99 in PDB code 4ATF (43)). This helix is not directly bordering the active site groove; however, it is immediately followed by a GH16_16-specific loop that contains a well-conserved tryptophan residue (Trp-109) constituting subsite −3. Another characteristic feature of GH16_16 is the C-terminal motif from residues 308 to 315 that also presents an α-helix providing a tryptophan that forms the +3 subsite. Opposite of this feature is a loop including residues His-215–Phe-222, which contains a strictly conserved arginine residue (Arg-219) that is involved in binding the 3,6-*anhydro* bridge of agarose in subsite −2.

##### GH16_17

GH16_17 contains κ-carrageenases (EC 3.2.1.83) from both Proteobacteria and Bacteroidetes. GH16_17 is the most distinct subfamily among those that hydrolyze marine carbohydrates, because it segregates at comparatively high *E*-value thresholds (Figs. 2 and 4). Examination of sequence subgroups in this subfamily highlights how sequence differences caused by speciation can give the appearance of further subfamilies without a functional basis. The SSN subclusters (Fig. 4) and phylogenetic clades (Fig. 5) correspond roughly to taxonomic subdivisions. Two members from different subbranches have been structurally and biochemically analyzed, indicating that subtle differences in substrate recognition and mode of action (perhaps even the lifestyle of the organism) are the result of evolutionary drift, whereas substrate specificity have remained constant; both are clearly κ-carrageenases (44).

Despite the observed phylogenetic divergence from the β-agarases (GH16_15 and GH16_16) and the β-porphyranases (GH16_12), subfamily GH16_17 contains a similar, characteristic N-terminal spatial arrangement (Fig. 5*B*). Otherwise, a key feature of this subfamily is vast diversity in which only few elements are strictly conserved. A notable differentiator is found in the loop that follows the conserved tryptophan comprising the −1 subsite, which contains a well-conserved tyrosine or phenylalanine (Tyr-143 in PDB code 5OCR) (44) that provides a hydrophobic environment to accommodate the 3,6-*anhydro* bridge in the −2 subsite. Importantly, a loop that is stabilized through two anti-parallel β-strands is positioned directly above the −1 subsite, thereby providing a strictly conserved arginine (Arg-263) to bind the κ-carrageenan–specific sulfate group on O4 of galactose residues. GH16_17 members have sequence variation around the positive subsites, indicating that subtle differences in substrate specificity might be found among this divergent subfamily.

##### GH16_18

GH16_18 is a large subfamily with 2,576 members. The subfamily is entirely composed of fungal enzymes including biochemically characterized chitin β(1,3)/β(1,6)-glucosyltransferases (EC 2.4.1.–) and cell-wall modifying enzymes (EC 3.2.1.–/2.4.1.–).

GH16_18 have a characteristic N terminus, starting with a disulfide bridge (residues 25–40 in PDB code 6IBW (unpublished)), which is arranged into a triple-stranded β-sheet with the C terminus. Strikingly, no residues from this loop appear to participate to substrate binding in the negative subsites. On the other hand, one strictly conserved tryptophan, Trp-207, forms a platform at the −2 subsite and the positive subsites also contain one strictly conserved tryptophan residue (Trp-221) and two largely conserved aromatic residues (Phe-137 and Tyr-145) that form large hydrophobic platforms to accommodate the substrate. Trp-221 is situated in a subfamily-specific α-helix, α1, which is the only true α-helix present in GH16_18 members. Although Phe-137 and Tyr-145 are not strictly conserved, the loop that contains these residues is characteristic and largely conserved within GH16_18 members.

##### GH16_19

GH16_19 derives as a sister clade to GH16_18 (Fig. 5*A*) and is composed of fungal enzymes, including a biochemically characterized chitin β(1,3)/β(1,6)-glucosyltransferase (EC 2.4.1.–) (45). Notably, many fungi have orthologs in both GH16_18 and GH16_19. Apart from statistically significant sequence differences in the GH16 module, a major difference between the two subfamilies is the presence of a CBM18 (predicted to bind chitin) in practically all enzymes of GH16_19, whereas no CBM is associated with GH16_18. No tertiary structural representatives currently exist in GH16_19.

##### GH16_20

GH16_20 is a well-characterized subfamily composed of plant enzymes specific for xyloglucan (46). Members of this subfamily are either xyloglucan *endo-*transglycosylases (XETs, EC 2.4.1.207) or xyloglucan *endo-*hydrolases (EC 3.2.1.151) (28, 47).

A significant key feature of GH16_20 is the addition of a large C-terminal domain (residues 232–264 in PDB code 2VH9 (48); InterPro and PFAM “XET_C”) that extends the active-site cleft at the positive subsites. In addition, a well-conserved loop region (residues 181–190) is located immediately adjacent to the catalytic residues and provides a strictly conserved tryptophan (Trp-185) that forms a hydrophobic platform at the +1 subsite. At the negative subsites, the loops bordering the active-site cleft are characteristically short in GH16_20 members (49). The resulting broadening of the active-site cleft appears to be responsible for the recognition of the highly branched xyloglucan chain (50, 51). One exception is the loop that precedes the β-strand containing the catalytic E*X*D*X*E motif, which is specifically lengthened in the xyloglucan *endo-*hydrolases (28). Notably, the aromatic platform of the −1 subsite in GH16_20 members is a tyrosine (Tyr-81), rather than a tryptophan found in most other GH16 members.

##### GH16_21

Historically known as the licheninase (EC 3.2.1.73) subfamily (30, 52), this subfamily has more than 30 biochemically characterized representatives among bacteria. Interestingly, a few members are found in the early diverging fungal lineage Chytridiomycota, including one biochemically characterized *endo*-β(1,3)/β(1,4)-glucanase (53). The *endo*-β(1,3)/β(1,4)-glucanases in GH16_21 strictly hydrolyze only the β(1,4)-glucosidic linkage in mixed-linkage β-glucan, typically at the anomeric position of backbone glucosyl units bearing a β(1,3)-glucan kink, and do not hydrolyze β-glucans containing only β(1,3)- or β(1,4)-linkages. Thus, GH16_21 are functionally different from the *endo*-β(1,3)/β(1,4)-glucanases found in GH16_3, which hydrolyze β(1,3)- or β(1,4)-linkages in mixed-linkage β-glucan, as well as β-glucans with only β(1,3)-linkages, such as laminarin.

Members of GH16_21 are among the shortest sequences, at ∼210 residues, whereas the average length of most of the other GH16 proteins is 240 residues. Consequently, characteristic features of this subfamily are short loops surrounding the substrate-binding groove. The conserved stretches are concentrated in four regions that border the central cleft, two on each side, which contain aromatic residues important for substrate binding (Tyr-24, Tyr-94, Trp-103, and Trp-192 in PDB code 1GBG) (54). Two of the characteristic loops contain short helical segments; the first (residues 91–100) is located at the −1 subsite, directly preceding the active site E*X*D*X*E motif, whereas the second borders the active site on the opposite side (residues 189–193), thereby providing a strictly conserved tryptophan at the +1 subsite. In addition, and similar to the GH16_20 subfamily, the aromatic platform at the −1 subsite in GH16_21 members is a phenylalanine (Phe-92), not a tryptophan.

### Uncharacterized subfamilies

Six well-defined subfamilies currently await definition of biochemical activity (Table 1 and Fig. 2). In particular, two very large subfamilies of fungal origin, the two sister subfamilies GH16_22 and GH16_23, which collectively contain ∼700 sequences, have so far gone unstudied. Likewise, two sister subfamilies, GH16_5 and GH16_7, limited to Proteobacteria, as well as GH16_6 with bacterial members, also remain unexplored. Noteworthy is the early diverging subfamily GH16_12, a sister clade to the newly discovered GH16_13 furcellaranases that, despite few members, has high taxonomic diversity (Figs. 2 and 5*A*).

### Nonclassified sequences

Roughly 3% of the analyzed GH16 sequences were not assigned to subfamilies (Fig. 2), primarily because of a lack of a sufficient number of orthologs in the CAZy database to define a subfamily with at least 20 members and sufficient taxonomical diversity. Among these is the only characterized GH16 member from a virus (*Paramecium bursaria* Chlorella virus 1, GenBank^TM^ accession no. AAC96462.1), which is an *endo*-β(1,3)/β(1,4)-glucanase that is distant from, but most closely related to, members of subfamily GH16_3. Other examples include two small groups related to the GH16_11 and GH16_12 β-porphyranase subfamilies, containing eight members and one biochemically characterized β-porphyranase each: β-porphyranase A (PDB codes 3ILF and 4ATE) (39, 43) and β-porphyranase C, respectively, from *Zobellia galactanivorans* DsijT. It is anticipated that these orphan sequences may seed additional subfamilies as the number of sequences in GenBank^TM^, from which the CAZy database is derived, continues to grow (7).

## Discussion

### Advantages and limitations of SSN-based subfamily classification

The utilization of a sequence similarity network–based approach allowed the division of 22,946 GH16 catalytic modules into subfamilies in a scalable, computationally efficient manner. Comparatively rapid generation of an all-*versus*-all pairwise scoring matrix, facile generation of SSNs at increasing BLAST *E*-value thresholds, and analysis of precision and recall rates guided the selection of an SSN cutoff value producing 23 robust subfamilies (Figs. 2 and 4*A*). A particular advantage of the SSN-based approach *versus* classical phylogenetic methods based on MSAs is the ability to utilize the full sequence data set without the need for down-sampling to reduce computation time.

For example, the previous division of GH5 (9) and GH43 (12) into subfamilies based on molecular phylogeny coped with the large amount of sequences (2,333 and 4,455, respectively) by employing the common practice of initial clustering of similar sequences, using algorithms such as UCLUST and CD-Hit (55, 56) to reduce the data sets. The clustering percentage identity limitations for UCLUST and CD-hit are 50 and 40%, respectively; thus, to obtain a reliable clustering, percentage identify cutoffs are usually set at 75% or higher (9, 12). In our preliminary analyses, applying a clustering cutoff of 75% to the 22,946 GH16 sequences yielded a reduced data set of 7,557 sequences, which is still an order of magnitude larger than the data set limitations for highly accurate MSA (57, 58) and subsequent maximum-likelihood phylogenetic tree estimation (59). Thus, a significant advantage of SSN generation is the superior computational efficiency caused by fundamental differences in algorithm complexity compared with phylogenetic approaches. This allowed us to analyze the entire, unreduced GH16 data set, which is 5, 10, and 13 times larger, respectively, than those used to classify GH43, GH5, and GH13 into subfamilies (9, 10, 12). Not least, a significant advantage of the combined BLAST-SSN approach is that it allows immediate recall of exact sequences from the data set, including their precise location within the SSN, at any time, whereas individual sequence information is lost in phylogenies based on representative sequences.

On the other hand, SSNs are unable to establish unambiguous evolutionary relationships between subfamilies. As observed for the SSN at *E* = 10^−55^ (Fig. 4*A*), which we use to define GH16 subfamilies, there is no intersubfamily connectivity, whereas at a relaxed threshold of *E* = 10^−25^, the SSN reveals only the most basic relationships (Fig. 4*B*). For example, GH16_17, which contains the marine carbohydrate-active κ-carrageenases, shows no connectivity to the other marine polysaccharidase subfamilies GH16_16, GH16_11, GH16_13, GH16_14, and GH16_15 at *E* = 10^−25^, whereas these subfamilies appear to be connected to more evolutionarily distant subfamilies (30), *e.g.* GH16_3 (comprising terrestrial *endo*-β(1,3)-glucanases and *endo*-β(1,3)/β(1,4)-glucanases; Fig. 4*B*). In contrast, a representative phylogenetic tree (Fig. 5*A*) clearly indicates that the κ-carrageenases form a sister clade to the other marine subfamilies, in agreement with a previously proposed evolution of GH16 diversity (30).

### A roadmap for functional glycogenomics

The delineation of large families such as GH16 into subfamilies can greatly improve predictive power to guide future functional analyses of individual family members, as has been previously exemplified for GH5 (9), GH13 (10), GH43 (12), and the polysaccharide lyase families (13). In particular, subfamily association can provide strong suggestions of likely substrates, or substrate families, that should be prioritized in biochemical assays. Not least, subfamilies with no, or very few, functionally characterized members hold significant untapped potential for biochemical discovery. Together, ongoing exploration of “known” and “unknown” subfamilies will continue to refine understanding of protein structure–function relationships across the evolutionary landscape of GH16.

In such endeavors, and especially for unsupervised bioinformatics, it is essential to bear in mind that this subfamily classification has certain predictive limitations. Sequence alignment–based approaches to delineate subfamilies, including both SSN and phylogenetic approaches, have insufficient resolution to segregate sequences differing by minor variations, which may nonetheless have large effects on biochemical and biological function. For example, it is well-known that single amino acid substitutions can switch substrate specificity in glycosidases (60, 61).

Within GH16 subfamilies, such limitations are exemplified by several cases. Neither SSNs (Fig. 4*A*) nor phylogeny (Fig. 5*A*) allow for the segregation of the β(1,3)-glucanases in GH16_4 from the homologous noncatalytic binding proteins, in which the catalytic residues are mutated, even at very high threshold values (*E* > 10^−85^; Figs. S1 and S2). GH16_3 is known to comprise both *endo*-β(1,3)-glucanases (laminarinases, EC 3.2.1.39) and *endo*-β(1,3)/β(1,4)-glucanases (the latter hydrolyzing the β(1,4)-bond in mixed-linkage glucan, EC 3.2.1.73) (62), which likewise do not segregate cleanly in SSNs nor phylogenies. Lastly, the canonical double-displacement mechanism of GH16 enzymes allows for both glycosyl transfer to water (hydrolysis, EC 3.2.1.–) and/or carbohydrate acceptor substrates (transglycosylation, EC 2.4.1.–) (63). The subfamily classification described here does not segregate transglycosylases from hydrolases in four fungal subfamilies (GH16_1, GH16_2, GH16_18, and GH16_19) and one plant subfamily (GH16_20) (28) (Table 1), indicating that the determinants of such specificities represent weak sequence signals masked by background sequence noise.

In light of current rapid increases in sequence data volume and a comparatively limited amount of experimental CAZyme characterization, there is significant potential for the propagation of inaccurate functional annotations caused by overconfident bioinformatic assignments. Consequently, this jeopardizes the usefulness of such annotations. We therefore advocate a conservative approach, in which functional predictions are abandoned altogether in (meta)genomic sequence annotation, in favor of simply designating all predicted proteins by their family and subfamily numbers, *e.g.* GH16_*n*.

### The evolution of structure–function relationships in GH16

At the highest level, this subfamily classification enables the evolution of major structural features to be mapped across GH16. Generally, variability within a subfamily is concentrated in the loops connecting the β-strands of the concave β-sheet (forming the active site groove) rather than in the N-terminal or C-terminal regions. In contrast, the termini typically vary substantially between subfamilies (Fig. 5*B*), *e.g.* the additional N- and C-terminal helices in GH16_1 or the expanded C terminus in GH16_20, which have significant functional ramifications (28).

Interestingly, some large subfamilies are highly conserved, such as the mycobacterial-specific GH16_9 subfamily and the plant-specific GH16_20 XTHs, whereas some smaller subfamilies, such as the GH16_16 β-agarases and GH16_17 κ-carrageenases, display substantial variability, even though they appear to display the same global substrate specificity (within the limits of current biochemical characterization). This might be related to specific constraints with respect to their biological functions. For example the crucial biological role of GH16_20 xyloglucan endo-transglycosylases and endo-hydrolases in plant growth and development (22, 46) might constrain sequence variations, whereas the bacterial catabolic enzymes may have diversified as a consequence of adaptation to available substrate diversity and environmental niches (2, 3, 64, 65). If this hypothesis holds true for the currently uncharacterized mycobacterial GH16_9 enzymes, a crucial biological role of the GH16 enzymes for these organisms can be expected.

### Looking to the future: emerging subfamilies

The CAZy database is derived exclusively from the NCBI GenBank^TM^ daily releases for practical reasons (7). Consequently, CAZy database, and thus the entire GH16 sequence set used here, does not capture sequences from nascent (meta)-genomic efforts, especially unfinished genomes from sequencing center databases (*e.g.* Joint Genome Institute, Broad Institute, Beijing Genomics Institute, etc.). Thus, it can be reasonably anticipated that the number of GH16 subfamilies will increase beyond the 23 presented here as the number of sequences in GenBank^TM^ continues to increase. This includes subfamilies from currently identified groups with fewer than 20 sequences or currently low taxonomic diversity, as well as newly emergent subfamilies from currently unexplored sequence space.

An example of an emerging GH16 subfamily is comprised of recently identified mixed-function *endo*-β(1,3)/β(1,4)-glucanases/*endo*-xyloglucanases from plants, for which biochemical and structural information exists (*e.g.* PDB code 5DZF and 5DZG) (51). These EG16 (endo-glucanase, GH16) members represent functional intermediates and an evolutionary link between the classic bacterial *endo*-β(1,3)/β(1,4)-glucanases in GH16_21 and the plant xyloglucan endo-transglycosylases and endo-hydrolases in GH16_20 (50, 51). A comprehensive census using genomes and transcriptomes of over 1,200 plant species has revealed a large collection of EG16 sequences in plant sequence databases, which are currently not deposited in GenBank^TM^ (23). Generation of SSNs including 717 plant EG16 orthologs with the 22,946 CAZy GH16 entries indicated that EG16 members segregate from GH16_20 at a threshold between *E* = 10^−35^ and *E* = 10^−40^ (data not shown) and thus will form an independent subfamily in the future. This subfamily was verified by maximum likelihood phylogeny, in which EG16 members constitute a sister group to the xyloglucan endo-transglycosylases and endo-hydrolases with high bootstrap support (Fig. 5*A*).

### CODA

Since the introduction of protein SSN analysis in its present form a decade ago (16), the use of SSNs has been growing in popularity for the analysis of large data sets (17, 66–74), in part because of a lower computational demand than classical molecular phylogeny. Here, we have utilized the power of SSN analysis to devise a robust subfamily classification of the large and diverse family GH16. This framework, which collates biochemical and structural data on characterized members, will enable more refined functional prediction to guide future bioinformatics and experimental studies. Nonetheless, we advocate a conservative approach to protein annotation, in which uncharacterized enzymes are referred to solely by their subfamily membership, to avoid the propagation of misleading functional annotation in public databases. To aid future sequence annotation, the GH16 subfamily classification is now publicly available in the CAZy database (http://www.cazy.org/GH16.html).^5^

## Experimental procedures

### Data acquisition

All GH16 members were extracted from the CAZy database (February 2018) (7) and used to retrieve amino acid sequences from GenBank^TM^. During this step, additional metainformation was gathered, including taxonomic lineage (kingdom, phylum, class, order, family, genus, and species ranks), modularity (presence of CBMs, signal peptides, etc.) of the full-length sequence (semimanually annotated using in-house CAZy pipelines) (75), and both biochemical and structural information from the literature. Sequences with less than 95% coverage to the GH16 family model were considered as fragments (13.7% in total) and not included in the final data set.

### Sequence similarity network analysis

All-*versus*-all pairwise local alignments of the 22,946 GH16 domain protein sequences were computed by BLAST+ 2.2.31 with default settings (specifically, scoring matrix: BLOSUM62; gap opening: 11; gap extension: 1) (76) using GNU Parallel (77), which generated the *E* value, bit score, alignment length, sequence identity, and sequence similarity for sequence pairs. The data were filtered using specific *E*-value threshold cutoffs (from least stringent, *E* = 10^−5^, to most stringent, *E* = 10^−120^) to generate a series of associated SSNs. To formally constitute a subfamily, connected clusters were required to contain at least 20 sequences, which ensured diversity above the taxonomic class level to mitigate against bias arising from over-representation of closely related organisms and GH16 homologs (9–13). Members of each putative subfamily were identified using NetworkX (78). SSNs were visualized with Cytoscape (79) using the yFiles organic layout. To simplify the display of large SSNs, nodes representing highly similar sequences (*E* value of 10^−85^) were merged into metanodes using the depth-first search algorithm (80). The bioinformatics workflow used here has been packaged into a graphical user interface-based program, SSNpipe, which is freely available on GitHub (https://github.com/ahvdk/SSNpipe).^5^

### Subfamily assessment/validation using hidden Markov models

Each SSN, defined by its clustering threshold (BLASTP *E* value), can be considered as a set of *N* assignments (*p* → *s*), where each of the 22,946 proteins, *p*, is assigned to its subfamily, *s*, among *S* total subfamilies. HMMs for each subfamily in each SSN were used to measure precision and recall rates to assess SSN utility and validate the choice of an optimal threshold value for GH16, as follows.

A library of *S*+1 HMMs was assembled, corresponding to one HMM for each subfamily *s* and an additional HMM for the remaining GH16 members. Each HMM was generated using the *hmmbuild* command in HMMER3.2 with default parameters (81). Sequence sets were first reduced in redundancy (75%) using UCLUST (55), the resulting sequences were aligned with MAFFT using the G-INS-i strategy (iterative refinement, using weighted sum-of-pairs and consistency scores, of pairwise Needleman-Wunsch global alignments) (82), and each alignment was inspected in Jalview (83) to manually define the boundaries of the GH16 module.

The *hmmscan* command in HMMER3.2 was then used to search the 22,946 GH16 modules against the collection of *S* + 1 HHMs. A protein *p*′ was considered to belong definitively to a subfamily HMM, *s*′, only if (i) the best-matching HMM *E* value was below 10^−30^ and (ii) the second best-matching HMM had an *E* value at least 10^−10^ fold greater (*i.e.* less significant). The resulting set of *P* predictions (*p*′ → *s*′) was compared with the *N* reference assignments (*p* → *s*) from the SSN. Identities between predictions and assignments were counted as true positives (*TP*). Predictions (*p*′ → *s*′) for a protein *p*′ not initially assigned to the same subfamily or to any subfamily (GH16 members unclassified in a subfamily by the SSN) were counted as false positives (*FP*). The assignments (*p* → *s*) for a protein *p* not predicted in any subfamily (GH16 unclassified at the subfamily level by the HMMs) are counted as false negatives (*FN*). To generate precision/recall plots, *precision* = *TP*/(*TP* + *FP*) and *recall* = *TP*/(*TP* + *FN*).

### Molecular phylogeny

For each subfamily, 30 random sequences (or all sequences in subfamilies with less than 30 members) were aligned with MAFFT using the G-INS-i (iterative refinement, using weighted sum-of-pairs and consistency scores, of pairwise Needleman–Wunsch global alignments) strategy (82). Three GH7 sequences (GenBank^TM^ accessions CAA37878.1, ABY56790.1, and AAM54070.1) were included as an out-group. The quality of the alignment was ensured by manual inspection in Jalview (83) and corrected according to available structural information if necessary. A maximum-likelihood phylogenetic tree was estimated with RAxML (84) (100 bootstrap replicates) and visualized with iTOL (85).

### Structural comparison

The crystal structure coordinates for 42 GH16 members were downloaded from the Protein Data Bank (PDB) and pairwise-superimposed starting from one of the shortest sequences (PDB code 1GBG) using the SSM algorithm (86) in Coot (87). One representative member was selected for those subfamilies where multiple structures are available (Table 1). For each subfamily, at least 10 randomly chosen sequences, in addition to that of the structural representative, were aligned with Multalin (88) and visualized adding the secondary structure elements using Espript (89). For each subfamily the superimposed coordinates were visually inspected for conserved and divergent residues around the active site groove, the central −1 and +1 binding subsites, and conserved and characteristic features were highlighted in structural icons using PyMOL (PyMOL Molecular Graphics System, version 2.0, Schrödinger).

## Author contributions

A. H. V., G. M., M. C., B. H., and H. B. conceptualization; A. H. V., G. M., M. C., B. H., and H. B. resources; A. H. V., N. T., V. L., G. M., M. C., B. H., and H. B. data curation; A. H. V., N. T., V. L., and G. M. software; A. H. V., N. T., V. L., M. C., B. H., and H. B. formal analysis; A. H. V., M. C., B. H., and H. B. supervision; A. H. V., M. C., B. H., and H. B. funding acquisition; A. H. V., N. T., V. L., M. C., B. H., and H. B. validation; A. H. V., N. T., V. L., G. M., M. C., B. H., and H. B. investigation; A. H. V., N. T., V. L., M. C., and H. B. visualization; A. H. V., N. T., V. L., G. M., M. C., B. H., and H. B. methodology; A. H. V., N. T., V. L., M. C., B. H., and H. B. writing-original draft; A. H. V., G. M., M. C., B. H., and H. B. project administration; A. H. V., N. T., V. L., G. M., M. C., B. H., and H. B. writing-review and editing.

## Supplementary Material

Supporting Information

## Footnotes

5 Please note that the JBC is not responsible for the long-term archiving and maintenance of this site or any other third party hosted site.

## Footnotes

4 The abbreviations used are:

CAZyme: carbohydrate-active enzyme
SSN: sequence similarity network
HMM: hidden Markov model
MSA: multiple sequence alignment
GH: glycoside hydrolase (family)
CBM: carbohydrate-binding module
PDB: Protein Data Bank
XET: xyloglucan *endo-*transglycosylase.
