# Supplementary material for: A subfamily roadmap of the evolutionarily diverse glycoside hydrolase family 16 (GH16)
Source: J Biol Chem. 2019 Sep 9;294(44):15973–86. doi: 10.1074/jbc.RA119.010619 (PMC6827312; doi:10.1074/jbc.RA119.010619)
Supplement: Supporting Information [file supp_294_44_15973__index.html]

A subfamily roadmap for functional glycogenomics of the evolutionarily diverse Glycoside Hydrolase Family 16 (GH16) — GH16 subfamilies — A subfamily roadmap of the evolutionarily diverse glycoside hydrolase family 16 (GH16) — GH16 subfamilies — Supporting Information 

# A subfamily roadmap of the evolutionarily diverse glycoside hydrolase family 16 (GH16)

## Supporting Information

- Supporting Information (to be published online) - Figures S1-S3.
