# Supplementary material for: A subfamily roadmap of the evolutionarily diverse glycoside hydrolase family 16 (GH16)
Source: J Biol Chem. 2019 Sep 9;294(44):15973–86. doi: 10.1074/jbc.RA119.010619 (PMC6827312; doi:10.1074/jbc.RA119.010619)
Supplement: Supporting Information [file supp_RA119.010619_155166_1_supp_390719_pxdmml.pdf]

Subfamily delineation of the evolutionarily diverse Glycoside Hydrolase Family 16 (GH16) provides a roadmap for functional glycogenomics

Alexander Holm Viborg[1,=], Nicolas Terrapon[2,3,=], Vincent Lombard[2,3], Gurvan Michel[4], Mirjam Czjzek[4], Bernard Henrissat[2,3,*], and Harry Brumer[1,5,6,7,*]

List of material included:

- Figure S1. SSN of 22,946 GH16 sequences at $E = 10^{-85}$.
- Figure S2. SSN of 22,946 GH16 sequences at $E = 10^{-120}$.
- Figure S3. Wall-eyed stereo representation of the superimposition of GH16 tertiary structures.

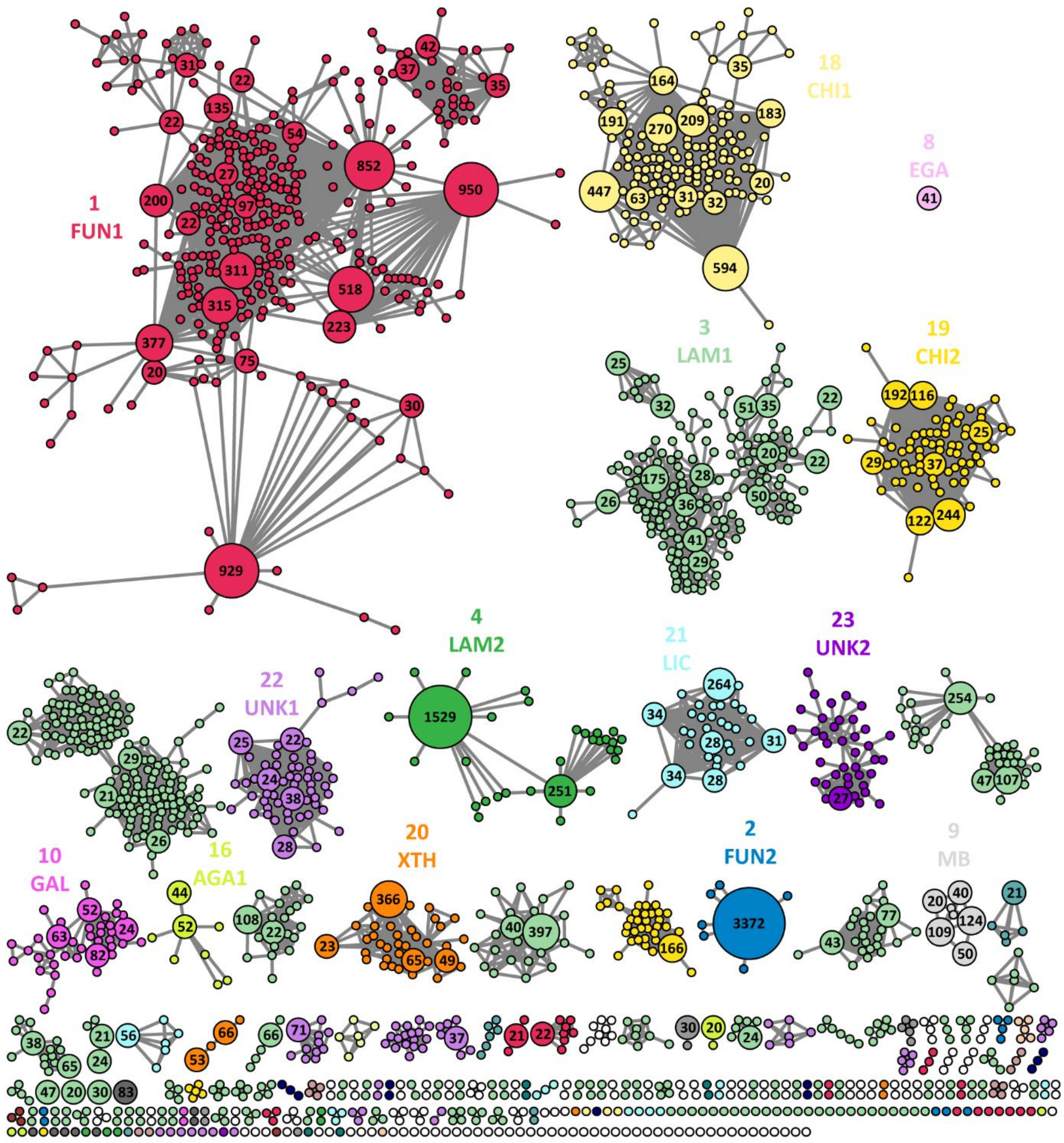

Figure S1. SSN of 22,946 GH16 sequences at $E = 10^{-85}$. Edges represent an *E*-value threshold below 10^{-85} (*cf*. Figure 4). Meta-nodes represent merged sequences at increased threshold ($E > 10^{-120}$); only meta-nodes containing 20 or more sequences are enlarged, with the number of merged sequences indicated. Each of the 23 defined subfamilies are indicated by a specific color (see Figure 2 for subfamily numbering and mnemonics). Not all subfamilies are labeled and only the biggest group of the segregated subfamilies is labeled. Non-classified sequences are indicated in white.

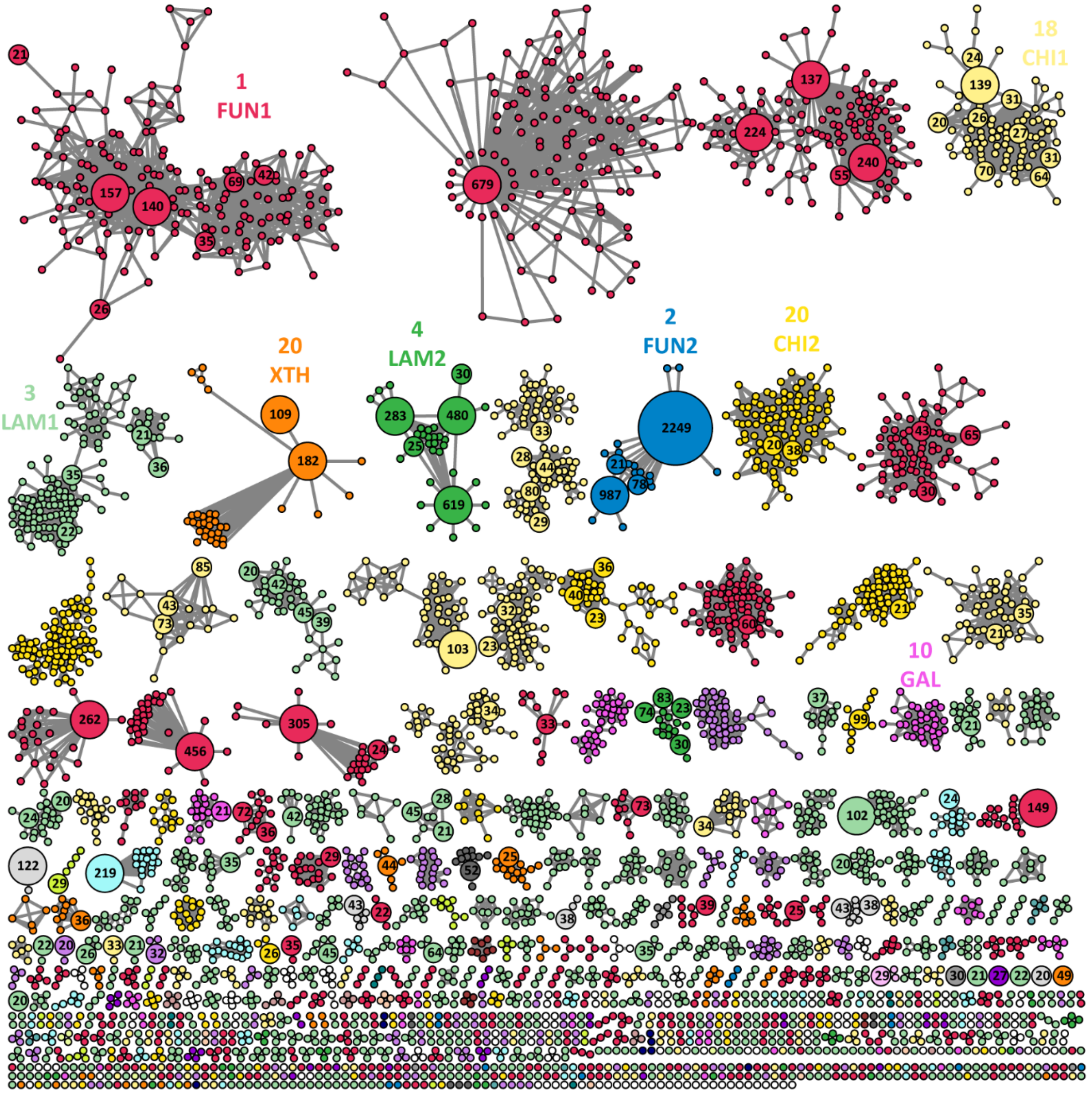

Figure S2. SSN of 22,946 GH16 sequences at $E = 10^{-120}$. Edges represent an E-value threshold below 10^{-120} (*cf.* Figure 4). Meta-nodes represent merged sequences at increased threshold ($E > 10^{-150}$); only meta-nodes containing 20 or more sequences are enlarged, with the number of merged sequences indicated. Each of the 23 defined subfamilies are indicated by a specific color (see Figure 2 for subfamily numbering and mnemonics). Not all subfamilies are labeled and only the biggest group of the segregated subfamilies is labeled. Non-classified sequences are indicated in white.

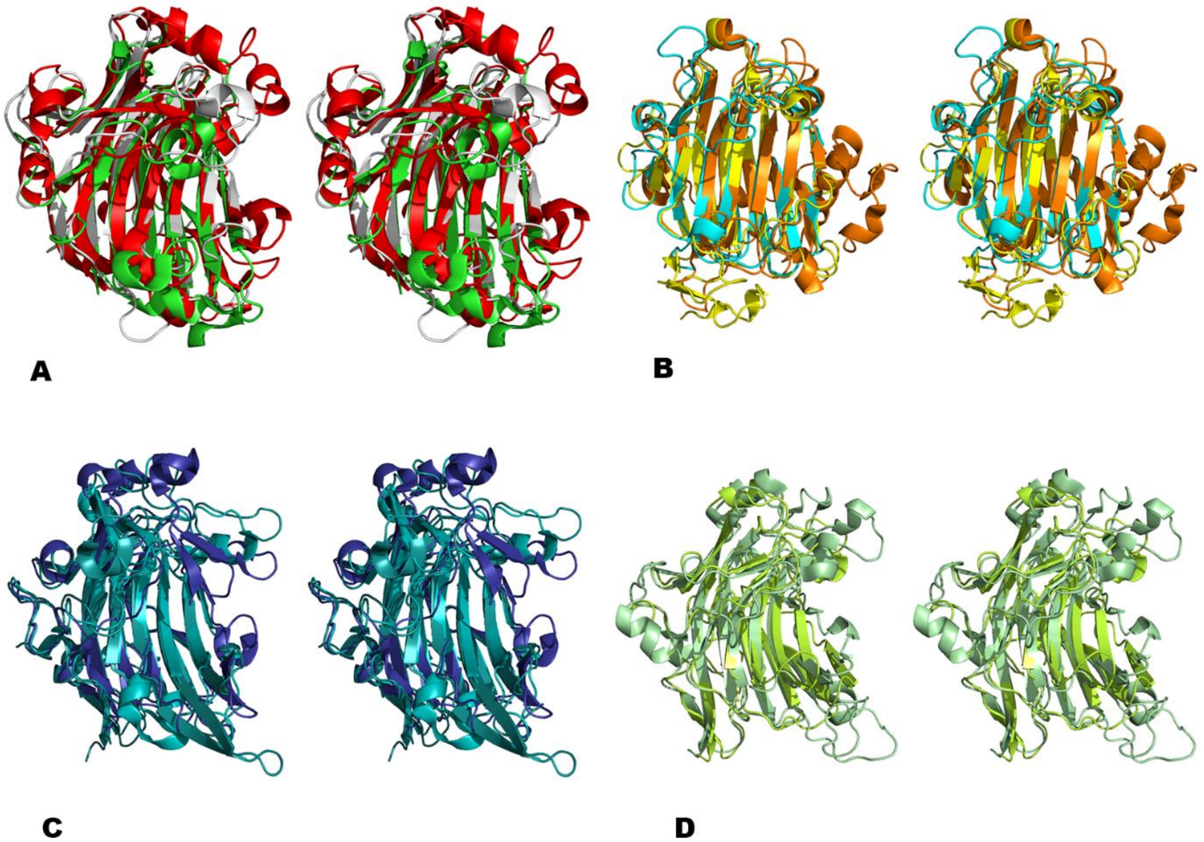

Figure S3. Wall-eyed stereo representation of the superimposition of GH16 tertiary structures. The 3D structures of members of neighboring subfamilies in the phylogenetic tree are superimposed using the secondary-structure-matching algorithm (SSM) implemented in COOT. The superimpositions highlight that despite the phylogenetic proximity of the members of these subfamilies, extensive differences are present, especially in the loops surrounding the active site groove. **A**. Superimposition of 2CL2 from GH16_1 (red), 4CTE from GH16_3 (light green) and 4PQ9 from GH16_9 (light grey). **B.** Superimposition of 5NDL from GH16_18 (light yellow), 2VH9 from GH16_20 (orange) and 1GBG from GH16_21 (cyan). **C.** Superimposition of 3JUU from GH16_11 (light steel), 4AWD from GH16_12 (steel) and 5OCR from GH16_17 (dark blue). **D.** Superimposition of 4ATF from GH16_16 (lime green) and 6HY3 from GH16_15 (pale lime green). The colors of the ribbon representations correspond to those of the respective group in the phylogenetic tree (Figure 5A).
